# Genomics of response to immune checkpoint therapies for cancer: implications for precision medicine

**DOI:** 10.1186/s13073-018-0605-7

**Published:** 2018-11-29

**Authors:** Jake R. Conway, Eric Kofman, Shirley S. Mo, Haitham Elmarakeby, Eliezer Van Allen

**Affiliations:** 1 Department of Medical Oncology, Dana-Farber Cancer Institute, Boston, MA 02215 USA; 2 Broad Institute of Massachusetts Institute of Technology (MIT) and Harvard, Cambridge, MA 02142 USA; 3 Department of Biomedical Informatics, Harvard Medical School, Boston, MA 02215 USA; 4 Department of System and Computer Engineering, Al-Azhar University, Cairo, 11751 Egypt

**Keywords:** Biomarkers, Cancer, Checkpoint, CTLA-4, Genomic, Immunotherapy, Inhibitor, PD-1, Response

## Abstract

Immune checkpoint blockade (ICB) therapies, which potentiate the body’s natural immune response against tumor cells, have shown immense promise in the treatment of various cancers. Currently, tumor mutational burden (TMB) and programmed death ligand 1 (PD-L1) expression are the primary biomarkers evaluated for clinical management of cancer patients across histologies. However, the wide range of responses has demonstrated that the specific molecular and genetic characteristics of each patient’s tumor and immune system must be considered to maximize treatment efficacy. Here, we review the various biological pathways and emerging biomarkers implicated in response to PD-(L)1 and cytotoxic T lymphocyte-associated antigen 4 (CTLA-4) therapies, including oncogenic signaling pathways, human leukocyte antigen (HLA) variability, mutation and neoantigen burden, microbiome composition, endogenous retroviruses (ERV), and deficiencies in chromatin remodeling and DNA damage repair (DDR) machinery. We also discuss several mechanisms that have been observed to confer resistance to ICB, such as loss of phosphatase and tensin homolog (PTEN), loss of major histocompatibility complex (MHC) I/II expression, and activation of the indoleamine 2,3-dioxygenase 1 (IDO1) and transforming growth factor beta (TGFβ) pathways. Clinical trials testing the combination of PD-(L)1 or CTLA-4 blockade with molecular mediators of these pathways are becoming more common and may hold promise for improving treatment efficacy and response. Ultimately, some of the genes and molecular mechanisms highlighted in this review may serve as novel biological targets or therapeutic vulnerabilities to improve clinical outcomes in patients.

## Background

**Fig. 1:**
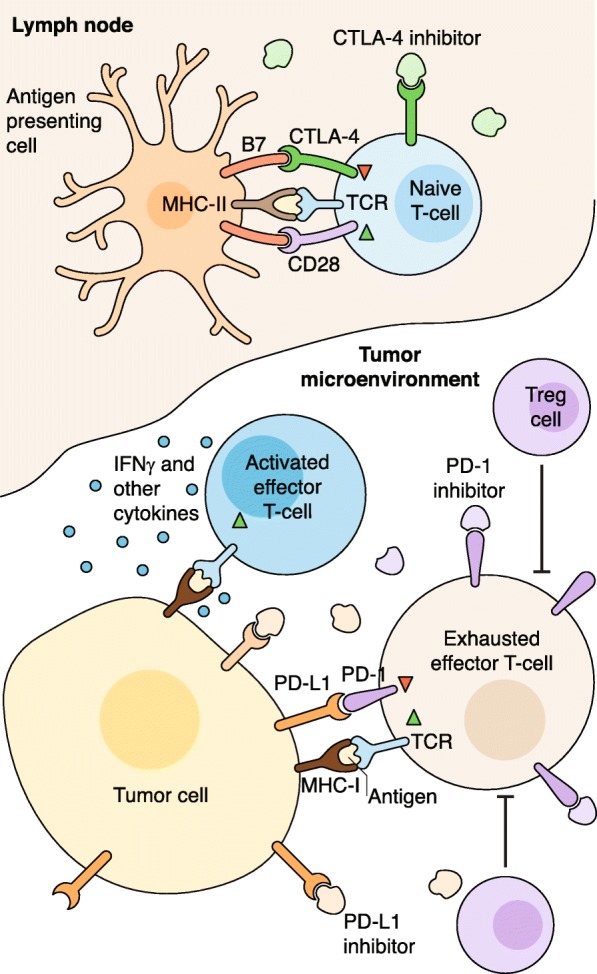
Immune checkpoint blockade. Professional antigen-presenting cells activate naive T cells through MHC-II complex/TCR and B7(CD80/86)/CD28 co-stimulatory binding. CTLA-4 inhibitors prevent competitive inhibitory binding of CTLA-4 with B7 ligands, which allows for more effective T cell activation. Activated effector T cells hone in on tumor cells and release IFNγ and other cytokines which boost the anti-tumor immune response. Tumor cells express PD-L1, which inhibits immune activity by binding to T cell PD-1 receptors, despite TCR recognition of target tumor antigens presented on tumor cell MHC-1 complex. Regulatory T cells (Tregs) also inhibit T cell activity and lead to an “exhausted” effector T cell phenotype. PD-1 inhibitors and PD-L1 inhibitors enhance the anti-tumor immune response by interrupting binding between tumor cell PD-L1 ligands and T cell PD-1 receptors. *CTLA-4* cytotoxic T lymphocyte-associated antigen 4, *MHC* major histocompatibility complex, *PD-1* programmed cell death protein 1, *PD-L1* programmed death ligand 1, *TCR* T cell receptor

Discovery of the immune checkpoints cytotoxic T lymphocyte-associated antigen 4 (CTLA-4) and programmed cell death protein 1 (PD-1) as key regulators of the adaptive immune response motivated the development of immune checkpoint blockade (ICB) therapeutics targeting these pathways. This therapeutics has caused a paradigm shift in the treatment of many forms of cancer. The targets of such therapies are CTLA-4 and PD-1 receptors, both expressed on the T cell surface, and PD-1 ligand PD-L1. In their natural capacity, CTLA-4 and PD-1 act as checkpoints that negatively regulate T cell activity to prevent escalated and chronic immune responses with deleterious autoimmune effects [1, 2]. However, the mechanisms by which CTLA-4 and PD-1 attenuate T cell activity differ considerably and occur at different stages in the T cell activation cycle. T cell activation is initiated when a T cell receptor (TCR) binds to an antigen presented on the major histocompatibility complex (MHC) of professional antigen-presenting cells (APCs), such as macrophages and dendritic cells (DCs). The activation process is completed through the interaction of co-stimulatory molecules CD28 on T cells and B7 ligands (CD80/86) on professional APCs [3] (Fig. 1).

A seminal study [4] revealed that CTLA-4 inhibits T cell activation by competing with CD28 for B7 ligands early in the adaptive immune response. This was confirmed by later work [5, 6] showing that CTLA-4 inhibits the initial stage of naïve T cell activation in the lymph nodes. In contrast to CTLA-4, which is constitutively expressed on T cells, PD-1 expression is contingent on T cell activation, and PD-1 is also expressed on B cells and natural killer (NK) cells [7, 8]. Inhibition of the immune response via PD-1 occurs upon its interaction with its corresponding ligands PD-L1 and PD-L2 [9]. PD-L1 is actively expressed on both APCs and tumor cells, suggesting that PD-1 inhibition is potentially effective at multiple steps in the immune response, both early on in the lymph nodes and later within the tumor microenvironment (TME) [10, 11]. PD-L2 has been studied less extensively than PD-L1, likely because PD-L2 is primarily upregulated on DCs and macrophages, which were thought to play a limited role in the TME [12].

In 2011, the US Food and Drug Administration (FDA) approved ipilimumab, an antibody that targets CTLA-4, for metastatic melanoma, making it the first FDA-approved ICB therapy for treatment of solid tumors [13]. In subsequent years, several antibodies targeting PD-1/PD-L1 have been approved by the FDA, including pembrolizumab (PD-1) for metastatic melanoma and a subset of non-small cell lung cancer (NSCLC) tumors, atezolizumab and durvalumab (PD-L1) for bladder cancer, and nivolumab (PD-1) for several malignancies [14–16]. Pembrolizumab is also FDA approved for tumors with mismatch repair deficiency, making it the first FDA-approved cancer drug based on genetics rather than tumor type or histology [17]. Currently, CTLA-4 and PD-1/PD-L1 inhibitors are the primary FDA-approved ICB therapies for solid tumors (Table 1).

**Table 1:** Approved immune checkpoint blockade therapies

Target	Drug	Company	Cancer type	Combination	Genomic and other indications	FDA approval date	References
PD-1	Nivolumab	Bristol-Meyers Squibb Company Inc.	Metastatic small cell lung cancer		Progression after platinum-based chemotherapy and at least one other line of therapy	August 16, 2018	[151]
			Metastatic colorectal cancer	Ipilimumab	Microsatellite instability-high (MSI-H) or mismatch repair deficient (dMMR)	July 10, 2018	[152]
			Untreated advanced renal cell carcinoma	Ipilimumab		April 16, 2018	[153]
			Melanoma	Adjuvant treatment	Involvement of lymph nodes	December 20, 2017	[154]
			Hepatocellular carcinoma			September 22, 2017	[155]
			Metastatic colorectal cancer		Mismatch repair deficient (dMMR) and microsatellite instability-high (MSI-H)	July 31, 2017	[156]
			Locally advanced or metastatic urothelial carcinoma			February 2, 2017	[157]
			Squamous cell carcinoma of the head and neck			November 10, 2016	[158]
			Classic Hodgkin lymphoma			May 17, 2016	[159]
			Advanced renal cell carcinoma			November 23, 2015	[160]
			Metastatic non-small cell lung cancer			October 9, 2015	[161]
			Metastatic melanoma	Ipilimumab	BRAF V600 wild type	September 30, 2015	[162]
			Metastatic squamous non-small cell lung cancer			March 4, 2015	[163]
			Unresectable or metastatic melanoma			December 22, 2014	[164]
	Pembrolizumab	Merck & Co, Inc.	Non-small cell lung cancer	Carboplatin and either paclitaxel or nab-paclitaxel		October 30, 2018	[165]
			Metastatic, non-squamous non-small cell lung cancer	Pemetrexed and platinum		August 20, 2018	[166]
			Primary mediastinal large B cell lymphoma			June 13, 2018	[167]
			Metastatic cervical cancer		Express PD-L1 (combined positive score ≥ 1)	June 12, 2018	[168]
			Gastric or gastroesophageal junction adenocarcinoma		Express PD-L1	September 22, 2017	[169]
			Solid tumors		Microsatellite instability-high (MSI-H) or mismatch repair deficient (dMMR)	May 23, 2017	[170]
			Urothelial carcinoma			May 18, 2017	[171]
			Metastatic non-squamous non-small cell lung cancer	Pemetrexed and carboplatin		May 10, 2017	[172]
			Classic Hodgkin lymphoma			March 14, 2017	[173]
			Metastatic non-small cell lung cancer		Express PD-L1	October 24, 2016	[174]
			Recurrent or metastatic head and neck squamous cell carcinoma			August 5, 2016	[175]
			Unresectable or metastatic melanoma			December 18, 2015	[176]
			Metastatic non-small cell lung cancer		Express PD-L1	October 2, 2015	[177]
			Unresectable or metastatic melanoma		Following ipilimumab and, if BRAF V600 mutation positive, a BRAF inhibitor	September 4, 2014	[178]
PD-L1	Atezolizumab	Genentech Oncology	Metastatic non-small cell lung cancer			October 18, 2016	[179]
			Locally advanced or metastatic urothelial carcinoma			May 18, 2016	[180]
	Durvalumab	AstraZeneca Inc.	Stage III non-small cell lung cancer			February 16, 2018	[181]
			Locally advanced or metastatic urothelial carcinoma			May 1, 2017	[182]
	Avelumab	EMD Serono, Inc.	Metastatic Merkel cell carcinoma			March 23, 2017	[183]
CTLA-4	Ipilimumab	Bristol-Meyers Squibb Company, Inc.	Metastatic colorectal cancer	Nivolumab	Microsatellite instability-high (MSI-H) or mismatch repair deficient (dMMR)	July 10, 2018	[152]
			Untreated advanced renal cell carcinoma	Nivolumab		April 16, 2018	[153]
			Cutaneous melanoma			October 28, 2015	[184]
			BRAF V600 wild-type, unresectable or metastatic melanoma	Nivolumab		September 30, 2015	[162]

In a study that compared response of PD-1 monotherapy (nivolumab) with CTLA-4 monotherapy (ipilimumab) in patients with untreated metastatic melanoma, patients receiving PD-1 blockade showed improved progression-free survival (PFS) along with less frequent immune-related adverse effects (irAEs) [18, 19]. This lower irAE rate can be attributed to the fact that PD-L1 is primarily expressed by tumors, such that any immune response is largely restricted to the TME. Conversely, CTLA-4 inhibits the immune response at an earlier stage in the lymph nodes, and so CTLA-4 blockade results in a more widespread effect that is nonspecific for tumor antigens [20].

Though PD-1 blockade has yielded expanded clinical benefit compared to CTLA-4 blockade, including FDA approval for several tumor types, patient response is heterogeneous and predicting response is not straightforward using current biomarkers such as PD-L1 expression and tumor mutational burden (TMB). In metastatic melanoma, NSCLC, and renal cell carcinoma, for example, patients with low PD-L1 expression and low TMB have also responded to PD-1 blockade, albeit at a much lower rate [21–23]. Generally, variation in response rates to PD-1 blockade across patients cannot be accounted for by the mean level of PD-L1 expression, highlighting the difficulty of generalizing predictive biomarkers to response [24]. As PD-1 blockade is dependent on T cell recognition of tumor antigens, it may prove ineffective in cases where T cells lack TCRs corresponding to tumor antigens, tumors fail to present antigens via their MHC, or there is a lack of tumor infiltrating lymphocytes (TILs) [25, 26]. Furthermore, even when tumor cells express PD-L1, this expression can be spatially heterogeneous within the tumor, allowing for the selection of less immunogenic subpopulations and the potential for resistance [27, 28]. TMB and PD-L1 expression are still widely studied and used for clinical stratification of patients [29, 30], but their limitations as predictors for response to ICB highlight the need for additional genomic biomarkers.

Alterations in highly regulated alternative pathways, such as chromatin remodeling and the urea cycle (UC), have also recently been found to affect response to ICB. Loss of function (LoF) mutations in the *BAF*/*PBAF* and *EZH2-PRC2* chromatin remodeling complexes confer sensitivity to ICB through upregulation of IFNγ-stimulated genes. Additionally, dysregulation of the UC, considered a hallmark of cancer, has been shown to introduce its own mutational spectrum that produces highly immunogenic neoantigens and increased sensitivity to ICB. Tumor extrinsic factors, such as the relative abundance of various gut microbiome bacterial strains or the expression levels of endogenous retroviruses (ERVs), also influence the response to ICB.

The differential effects of cancer-related genes and pathways on the immune system can be leveraged for combination therapy with ICB. For example, mitogen-activated protein kinase (MAPK) inhibition in preclinical mouse models has been observed to increase TILs, IFNγ production, and MHC-I expression, and combination with ICB may be more efficacious than monotherapy. Mechanisms underlying resistance to ICB therapy also need to be considered. For instance, loss of the phosphatase and tensin homolog (PTEN), a common event in glioblastoma, prostate cancer, breast cancer, and melanoma, as well as indoleamine 2,3-dioxygenase 1 (IDO1) expression in the TME of soft tissue sarcomas, elicit resistance to ICB. In general, advances in mechanistic understanding of response and resistance to ICB and the predictive genomic biomarkers discussed in this review may influence treatment decisions and options. The specific molecular and genetic traits of each tumor should be considered in a cancer type-dependent manner to maximize ICB efficacy.

## Genome-wide determinants of response and resistance

**Fig. 2:**
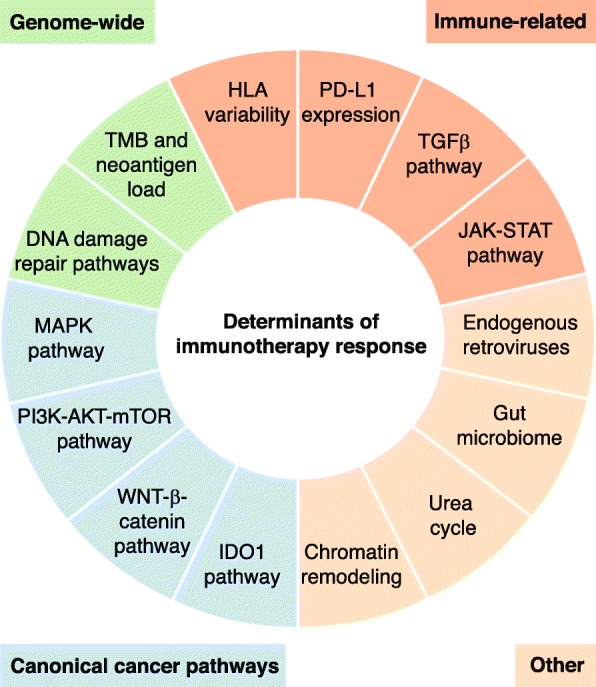
Pathways, genomic characteristics, and molecular mechanisms implicated in response to immune checkpoint therapy. Alterations in canonical cancer pathways such as the MAPK, PI3K, and WNT-β-catenin pathways are associated with increased resistance to ICB. Inactivation of the MAPK and PI3K pathways, through alterations such as PTEN loss, are associated with a reduction in TILs and decreased expression of pro-inflammatory cytokines in the TME. Conversely, activation of the WNT-β-catenin and IDO1 pathways results in suppression of T cells and NK cells in the TME. Genome-wide characteristics, including deficiencies in DNA repair machinery and increased tumor mutational/neoantigen burden, are also associated with resistance. Increased mutational burden has been shown to lead to an elevated neoantigen burden, which results in a highly immunogenic tumor. If the neoantigens are clonal, T cell response is capable of eradicating the entire tumor, rather than a subpopulation of tumor cells. Furthermore, decreased HLA variability, LoF alterations in the JAK-STAT pathway, and induction of TGFβ increase resistance to immune checkpoint therapy through alteration of the immune response directly. *HLA* human leukocyte antigen, *ICB* immune checkpoint blockade, *IDO1* indoleamine 2,3-dioxygenase, *JAK-STAT* janus kinase/signal transducers and activators of transcription*, LoF* loss of function, *MAPK* mitogen-activated protein kinase, *NK* natural killer, *PI3K* phosphoinositide 3-kinase, *PTEN* phosphatase and tensin homolog, *TGFβ* transforming growth factor beta, *TIL* tumor infiltrating lymphocytes, *TMB* tumor mutational burden

High TMB, along with the associated high neoantigen load it implies, can be predictive of increased T cell activity and an enhanced ICB response. Mutations in DNA damage repair (DDR) pathways can also be informative, with certain DDR signatures linked to high TMB. Importantly, these distinguishing tumor features are most predictive of ICB response when clonal, such that intratumor heterogeneity (ITH) must be incorporated into their assessment as genomic biomarkers (Fig. 2).

### Tumor mutational burden and neoantigen load

TMB and neoantigen load were among the earliest biomarkers of clinical response to ICB and remain widely used. Snyder et al. [31] first showed that higher TMB was associated with response to CTLA-4 therapy in metastatic melanoma, which was subsequently validated by Van Allen et al. [32]. Increased TMB is also associated with response to PD-(L)1 blockade. In desmoplastic melanoma, a rare melanoma subtype that has significantly higher TMB than cutaneous melanomas, Eroglu et al. [33] observed an exceptional objective response rate (ORR) of 70%, with 32% of patients exhibiting complete response. This response rate is among the highest to PD-(L)1 blockade for all cancer types [34, 35]. A higher TMB has been shown to correlate with an increase of cancer neoantigens presented via MHC on cancer cells, which is expected to result in increased levels of TILs [21, 25]. Though a vast majority of tumor-specific neoantigens are predicted to originate from subclonal passenger mutations, Miao et al. [36] identified 871 predicted driver neoantigens across 249 tumors in a pan-cancer cohort, eight of which were clonal and occurred recurrently in patients with complete or partial response. These results suggest that T cell response could potentially target all tumor cells.

Despite this, in a separate study, Van Allen et al. [32] leveraged pretreatment transcriptomic data to filter for putative neoantigens, and found that no single neoantigen sequence predicted response to CTLA-4 therapy. Thus, larger cohorts will be required to detect statistically significant associations between individual neoantigens and response. Although the connection between TMB and neoantigen load provides a biological explanation underlying ICB response in TMB-high tumors, TMB alone does not reliably predict response in all patients [37–39]. Identification of additional genomic factors that influence response are imperative to better understand and predict patient outcomes and refine therapeutic strategies.

### DNA damage repair pathways

Tumors with deficiencies in DDR pathways are less efficient at correcting genetic lesions and are accordingly associated with an increased TMB, neoantigen load, and better response to ICB [40–43]. In metastatic NSCLC, Rizvi et al. [21] first reported that three TMB-high ICB responders had tumors harboring deleterious mutations in several DNA repair and replication genes, including *POLD1*, *POLE*, and *MSH2*. Analogously, in a metastatic melanoma cohort, Hugo et al. [25] noticed significant enrichment of mutations in the homologous recombination (HR) repair gene *BRCA2* in PD-1 blockade responders compared to non-responders. This observation was corroborated in ovarian cancer, with tumors harboring *BRCA1*/*2* alterations having an increased predicted neoantigen load [44]. More recently, a trial of advanced urothelial cancers found that tumors with alterations in DDR pathways responded to ICB at higher rates than DDR wild-type tumors [45].

Identification of mismatch repair (MMR) deficiencies across 13 tumor types further solidified the significance of genomic alterations in DDR genes as a generalizable biomarker for immunotherapy response [17, 46]. Le et al. [46] found that patients with germline alterations in *MSH2*, *MSH6*, *PMS2*, and *MLH1*, consistent with either sporadic MMR-deficient tumors or Lynch syndrome, had similar ORR. Moreover, this similarity held across colorectal, endometrial, gastroesophageal, pancreatic, and prostate cancers. Of note, mutational signatures consisting of trinucleotide substitution patterns generated by underlying mutational processes, such as MMR and HR deficiency, may serve as a proxy for identifying DDR deficiency status in tumors prior to treatment with ICB [47, 48]. Furthermore, certain signatures associated with increased mutational load, such as MMR deficiency and UV mutagenesis, may also serve as a proxy for elevated TMB, whereas others, such as HR deficiency, may serve as a proxy for genomic instability.

### Tumor heterogeneity

The effect of ITH on the neoantigen landscape offers an additional explanation for the variability in ICB responses. McGranahan et al. [49] found increased sensitivity to both PD-1 and CTLA-4 blockade, and improved overall survival (OS), among NSCLC and melanoma patients with tumors harboring low ITH and high clonal neoantigen burden. Riaz et al. [50] confirmed this finding in a cohort of advanced melanoma patients and reported that higher pretreatment clonal TMB and lower subclonal TMB were associated with increased OS and response to nivolumab. As ITH increases, so does the chance that a tumor contains subclones able to evade the immune system and survive ICB therapy.

## Canonical cancer pathways implicated in response and resistance

Confirming the intricate relationship between immune response and tumor progression, alterations in several canonical oncogenes and tumor suppressors have also recently been associated with response to ICB. The majority of these genes function in the MAPK, PI3K-AKT-mTOR, and WNT-β-catenin pathways, all of which are firmly established as oncogenic signaling pathways with longstanding biological evidence for relevance to tumor formation and evolution. As several of these genes are targets of known inhibitors, any significant associations between these genes and ICB response may be leveraged to inform combination therapies of ICB with such inhibitors.

### MAPK pathway

The MAPK pathway is involved in a number of diverse cellular processes such as proliferation, differentiation, motility, apoptosis, and survival, and its oncogenic role has been well documented [51]. An emerging body of evidence has also identified a role for the MAPK pathway in regulating immune response in the TME. In mouse models, across various cancer histologies, inhibition of the pathway using MAPK/ERK (extracellular signal–regulated kinase) inhibitors (MEKi) resulted in enhanced TILs, IFNγ production, and MHC-I expression, suggesting that combination therapy of MEKi with PD-(L)1 or CTLA-4 blockade may improve response in patients with genomic alterations in the MAPK pathway [52–54]. Interestingly, the MAPK pathway is essential for T cell activation, proliferation, function, and lymphocyte survival, suggesting MEKi might simultaneously impede conventional T cell response [55]. Work in colon carcinoma mouse models has further demonstrated MEKi to be a double-edged sword: MEKi potentiates T cells in the TME by hindering TCR-driven apoptosis while inhibiting T cell priming in the lymph nodes [56]. However, Dushyanthen et al. [57] demonstrated that the T cell agonist antibodies α-4-1BB and α-OX-40 can rescue the adverse effects of MEKi in both mouse and human T cells, but this is dependent on activation of the downstream p38 and JNK pathways.

Co-mutation with MAPK pathway genes is also associated with response to ICB in a cancer type-dependent context. *KRAS*, a MAPK pathway gene, is one of the most frequent oncogenic drivers in lung adenocarcinoma (LUAC), and its co-mutation with *STK11* or *TP53* defines genomic subtypes with distinct mutational landscapes and immune profiles [58]. *KRAS*-mutant LUACs with *STK11* mutations experience significantly worse ORR, PFS, and OS compared to LUACs harboring only *KRAS* mutations. Mutations in *STK11* are also significantly associated with PD-L1 negativity in LUACs with intermediate-to-high TMB, regardless of *KRAS* mutation status, providing a biological explanation for the low response rate in *KRAS*/*STK11*-mutant LUACs. Conversely, *KRAS*-mutant LUACs with mutations in *TP53* exhibit an increased ORR and have similar PFS and OS to *KRAS*-mutant only LUACs [58, 59]. Thus, understanding the co-mutation patterns of driver genes in canonical cancer pathways, such as MAPK, may reveal novel relationships that inform response or resistance to ICB.

### PI3K-AKT-mTOR pathway

The PI3K-AKT-mTOR pathway is a key signal transduction system comprising several oncogenes and is involved in essential cellular processes such as cell survival, proliferation, and differentiation. The negative regulatory protein PTEN functions as a tumor suppressor by dephosphorylating PIP3, a key initiator of the PI3K-AKT-mTOR pathway [60, 61]. Loss of PTEN thus results in a constitutively activated PI3K-AKT-mTOR pathway and, consequently, an aberrant growth phenotype [62].

Recent studies have indicated that, in addition to its oncogenic effects, loss of PTEN leads to decreased effector T cell activity in the TME. In prostate mouse models, Toso et al. [63] found that Pten^−^ null mice exhibited high levels of infiltration by granulocytic myeloid-derived suppressor cells, which act to exclude CD8+ and NK cells from the TME and reduce their cytotoxic activity. Supporting this finding, Peng et al. [64] demonstrated that PTEN loss in melanoma cell lines and clinical samples was correlated with increased expression of vascular endothelial growth factor (VEGF) cytokines, which recruit regulatory T cells (Tregs) and other suppressive immune cells to render the TME less permeable to CD8+ effector T cells.

Such reduction in TILs would preclude an effective immune response even in the presence of checkpoint inhibitors, pointing to PTEN loss as a potential indicator of resistance to such therapies. Indeed, the potential ramifications of PTEN loss for successful checkpoint inhibition therapy were illuminated when George et al. [65] showed that the sole resistant metastatic site in a uterine leiomyosarcoma patient, otherwise extremely responsive to a PD-L1 inhibitor, had experienced biallelic *PTEN* loss.

It is well-established that *PTEN* loss, inactivation, or attenuation is a common genetic feature in multiple cancers, with *PTEN* loss of heterozygosity (LoH) found in more than a quarter of glioblastomas, prostate cancers, breast cancers, and melanomas [66]. Thus, the recent insights regarding the effect of *PTEN* on response to ICB could make it a widely informative biomarker for therapeutic decision-making.

### WNT–β-catenin pathway

WNT–β-catenin signaling is an evolutionarily conserved growth pathway that plays an essential role in both embryonic development and tissue maintenance in adults, regulating many biological processes, including homeostasis, hematopoiesis, and apoptosis [67]. Aberrancies in this pathway have been linked to many cancer types, including colorectal cancer, leukemia, melanoma, and breast cancer [68–70].

The role of the WNT pathway in cancer development has received a lot of attention, but its interaction with the immune system is also crucial. Spranger et al. [71] showed that T cell infiltration in the TME is inversely associated with intrinsic β-catenin signaling in metastatic melanoma patients. Using gene expression analysis to classify metastatic patients into T cell inflamed and non-T cell inflamed subtypes, Spranger et al. [71] found that non-T cell inflamed tumors were enriched with active β-catenin signaling. Mouse models validated this claim: mice constitutively expressing β-catenin exhibited significantly reduced TILs and increased resistance to ICB. Moreover, Spranger et al. [71] demonstrated that β-catenin suppresses chemokines needed to recruit DCs to the TME, resulting in reduced T cell priming.

The WNT–β-catenin pathway also influences recruitment of T cells to the TME through regulation of its downstream target, DKK2. In a recent study, Xiao et al. [72] described how high expression of DKK2 led to the suppression of T cells and NK cells in the TME. Indeed, DKK2 inhibition combined with PD-1 blockade in preclinical mouse models enhanced NK cell and CD8+ T cell cytotoxicity. These findings suggest that alterations known to activate β-catenin signaling should be considered prior to treatment with ICB.

### IDO1 pathway

Indoleamine 2, 3-dioxygenase 1 (IDO1) is an interferon-inducible immune checkpoint that converts tryptophan into kynurenines and is associated with immunosuppression in tumors [73]. Accumulation of kynurenines promotes activation of several pathways, including the PI3K-AKT-mTOR pathway [73, 74]. In a phase II clinical trial testing the combination of pembrolizumab and metronomic cyclophosphamide in 57 soft tissue sarcoma patients, Toulmonde et al. [75] observed tumor shrinkage in just three patients with only one experiencing a partial response, despite over 40% of cases expressing PD-L1 in the TME. Further evaluation found that the majority of sarcomas were infiltrated by M2 macrophages that expressed IDO1, which may explain the lack of response to PD-(L)1 inhibition [73–75]. Unlike M1 macrophages, which are activated through the IFNγ pathway, M2 macrophages are activated via interleukin (IL)-4 and IL-13 expression and are associated with the secretion of distinct cytokines (for example, TGFβ) and chemokines (for example, CCL17, CCL22, CCL24). Expression of IDO1 in the TME evidently limits the activity of PD-(L)1 blockade in a subset of cancers, and activation of this pathway should be tested for prior to administering PD-(L)1 therapy.

## Immune-related pathways involved in response and resistance

**Fig. 3:**
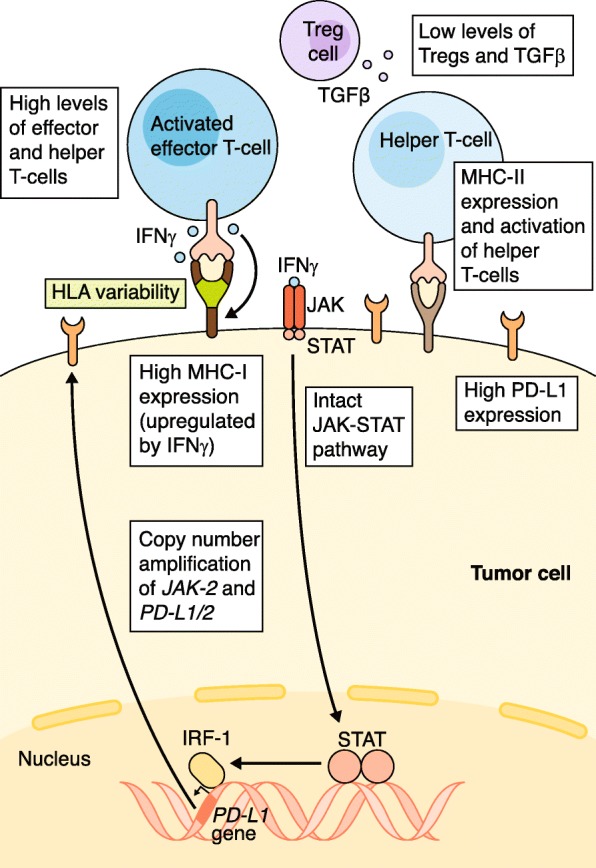
Immune-related features and pathways predictive of response to immune checkpoint blockade. Copy number amplifications of the *JAK-2*/*PD-L1/2* regions, increased PD-L1 expression via an intact JAK-STAT pathway culminating in IRF-1 binding to the *PD-L1* promoter, high MHC-I/II expression, and HLA variability all correlate with response to ICB. Elevated concentrations of effector and helper T cells and low concentrations of Tregs and TGFβ in the TME are also associated with response to ICB. *HLA* human leukocyte antigen, *ICB* immune checkpoint blockade, *IRF-1* interferon regulatory factor 1, *JAK-STAT* janus kinase/signal transducers and activators of transcription*, MHC* major histocompatibility complex, *PD-L1* programmed death ligand 1, *TGFβ* transforming growth factor beta, *TME* tumor microenvironment*, Treg* regulatory T cell

Variability in immune-related pathways also, naturally, affects response to immunotherapy. There has been increased interest in exploring the mechanisms regulating IFNγ propagation and expression of PD-L1 and MHC, especially with respect to the Janus kinase/signal transducers and activators of transcription (JAK-STAT) pathway, human leukocyte antigen (*HLA*) genes, and transforming growth factor beta (TGFβ) levels. As the downstream effects of these pathways on immune response become clearer, alterations in their comprising genes may help to categorize patients based on the likelihood of ICB response (Fig. 3).

### HLA variability

The MHC-I complex aids in the presentation of cancer neoantigens to CD8+ cells, and variability among the genes encoding it, including *B2M* and the *HLA-I* genes (*HLA-A*, *HLA-B*, and *HLA-C*), has been demonstrated to influence ICB response. Zaretsky et al. [76] reported a treatment-resistant melanoma case possibly explained by a truncation of *B2M*, which is involved in antigen presentation. A similar case report of a resistant colorectal tumor found LoH in the HLA-C*08:02 region of tumor cells, which is required for KRAS G12D neoantigen presentation [77]. More generally, phylogenetic analysis by McGranahan et al. [78] found that *HLA* LoH alterations are under positive selection in NSCLC tumors. Conversely, increased heterozygosity at *HLA-I* loci was associated with better survival among advanced cancer patients undergoing ICB, with certain supertypes such as HLA-B44 experiencing significantly better OS than others (for example, HLA-B62) [79]. The focal nature of *HLA* LoH, its enrichment in metastatic sites, and subclonal frequencies suggest it may play an important role as a mechanism of immune escape.

Adding another layer of complexity, the MHC-II complex (encoded by *HLA-DP*, *HLA-DM*, *HLA-DO*, *HLA-DQ*, and *HLA-DR*) is canonically expressed by professional APCs to present antigens to CD4+ cells, but has also been found to be expressed by some tumor cells and to have an effect on ICB outcomes [80]. An analysis of a classic Hodgkin lymphoma cohort found that increased PD-L1 and MHC-II expression on malignant Hodgkin Reed-Sternberg cells correlated with better PFS in response to PD-1 blockade. Interestingly, Hodgkin Reed-Sternberg cells lacked MHC-I expression in 92% of complete responders [81]. This suggests an alternative mechanism for ICB via CD4+ cell recognition of tumor antigens. Further highlighting the complex relationship between HLA variability and ICB response, Rodig et al. [82] recently reported differential effects of response for MHC-I/II in advanced melanoma patients, with MHC-I and MHC-II expression correlating with anti-CTLA-4 and anti-PD-1 efficacy, respectively. T cells are more likely to be primed for activation in the thymus as a result of CTLA-4 blockade, but their cytotoxic activity still relies on tumor MHC-I expression. Meanwhile, in the absence of tumor MHC-I expression, MHC-II expression functions as a complementary means of activating T-helper cells. This synergistic relationship justifies anti-PD-(L)1/anti-CTLA-4 combination therapy and highlights the importance of assessing pre-treatment expression levels for both MHC complexes.

### JAK-STAT pathway

The JAK-STAT family of signaling pathways has long been known to play important roles in several immunological functions, with established links between *JAK-STAT* germline mutations and immune-related diseases [83]. The particular implications of the JAK-STAT pathway in immunotherapy revolve around its role in propagating IFNγ.

Expression of IFNγ results in the upregulation of MHC expression [84], which increases the likelihood of neoantigen presentation in tumor cells and further boosts anti-tumor immune response. However, IFNγ is a double-edged sword. Separate 2017 studies by Ribas et al. [83] and Moon et al. [85] showed that IFNγ exposure in gastric cancer and melanoma cell lines also leads to increased expression of PD-L1 via the JAK-STAT pathway. This immune escape mechanism was corroborated by findings in gastric and ovarian cancer cell lines that stromal CD8+ infiltration levels are correlated with both IFNγ levels and tumor PD-L1 expression [86, 87]. Thus, somewhat counterintuitively, TIL secretion of IFNγ can itself induce a negative feedback loop and adaptive resistance by upregulating PD-L1 on tumor cells.

Because IFNγ exposure results in the upregulation of PD-L1, PD-(L)1 blockade therapies are most efficacious when the JAK-STAT pathway is intact or even potentiated. Amplification of chromosomal region 9p24.1, which includes the genes *PD-L1*, *PD-L2*, and *JAK2*, was recently found to be a biomarker for high anti-PD(L)-1 response rate in Hodgkin lymphoma [88]; expression of PD-L1 is augmented in this case, not only directly via amplification of *PD-L1* itself, but also indirectly through a more active JAK-STAT pathway. Meanwhile, Manguso et al. [89] demonstrated via an in vivo CRISPR knockout screen that tumors lacking key elements of the JAK-STAT pathway failed to upregulate MHC-I molecules and were consequently better able to evade immune surveillance. A study of four melanoma patients who experienced relapses following PD-L1 blockade therapy bolstered this finding, as two of the four resistant tumors harbored JAK1 or JAK2 inactivating mutations [90].

The loss of IFNγ-mediated JAK-STAT signaling has also been shown to contribute to resistance to CTLA-4 blockade. In a cohort of melanoma patients receiving ipilimumab, Gao et al. [91] found that tumors responding poorly to treatment were enriched for copy number alterations in IFNγ pathway genes compared to responders, including loss of the interferon gamma receptor 1 gene (*IFNGR1*) and *JAK2*, and amplification of pathway inhibitors such as *SOCS1*. Although most of the literature since has focused primarily on *JAK1* and *JAK2*, Van Allen et al. [32] found that activating somatic and germline mutations in *JAK3*, which is associated with increased PD-L1 expression in a lung cancer cell line, potentially explained dramatic and repeated responses to PD-L1 blockade in a patient with metastatic LUAC [92]. Similarly, a CRISPR screen aimed at discovering LoF mutations conferring resistance to ICB identified a novel function for the gene *APLNR* in modulating the JAK-STAT pathway signal [93].

In 2017, Shin et al. [90] suggested that the presence of *JAK1/2* LoF mutations can be a biomarker for resistance to PD-L1 therapy and that patients whose tumors exhibit such mutations would be poor candidates for ICB. Additionally, Luo et al. [94] recently reported that *JAK1* plays a more indispensable role than *JAK2* in IFNγ-induced expression of MHC and PD-L1. Though few specific actionable *JAK-STAT* variants have been pinpointed, it is clear that any major alterations to this pathway are likely to affect responses to PD-(L)1 and CTLA-4 blockade.

### TGFβ pathway

TGFβ is a cytokine involved in the regulation of development, growth, inflammation, and wound healing, among other biological processes. In the context of cancer, TGFβ has been found to promote an immunosuppressive TME, enhancing the function of Tregs while damping down the activity of cytotoxic lymphocytes and NK cells [95]. Results from recent studies show that levels of TGFβ may serve as a prognostic biomarker for the efficacy of ICB. In a mouse model of colorectal cancer, TGFβ promoted T cell exclusion and a “cold” TME phenotype, and its inhibition led to an enhanced immune response when co-administered with anti-PD-L1 [96]. Similar effects were described in a metastatic urothelial cancer cohort where the TME of non-responders had high levels of TGFβ [97]. Inhibition of TGFβ signaling in conjunction with ICB may be one method of increasing the efficacy of immunotherapy in tumors with an elevated concentration of TGFβ.

In summary, variation in *HLA* genes and the expression levels of the MHC I/II complexes can shape the anti-tumor response by modulating recognition of tumor antigens by the adaptive immune system. Simultaneously, variation in the JAK-STAT pathway modulates IFNγ and PD-L1 expression levels and consequently TIL cytolytic activity, with high levels of TGFβ potentially hampering this activity. Any alteration disrupting the complex interaction of these pathways can enable tumor immune escape. Therefore, the JAK-STAT pathway, TGFβ pathway, and HLA variability should be analyzed jointly when considering their effect on ICB response.

## Other molecular factors affecting response and resistance

Several pathways not traditionally studied in cancer genomics have been implicated in response to ICB. LoF alterations in chromatin remodeling complex genes are associated with resistance to ICB, while elevated expression of endogenous retroviruses and dysregulation of the urea cycle are associated with response. In addition, the relative abundance of certain microbiome species is associated with both response and resistance to ICB.

### Chromatin remodeling

Several recent studies have demonstrated a role for chromatin regulation in response to PD-(L)1 and CTLA-4 blockade. The BRG1-associated factor (BAF) and polybromo-associated BAF (PBAF) complexes, which function as both chromatin remodelers and tumor suppressors, are mutated in more than 20% of human cancers [98–101]. Although both complexes share core subunits, the BAF complex uniquely contains ARID1A/B, whereas the PBAF complex uniquely contains ARID2, PBRM1, and BRD7. Both clinical and pre-clinical models have revealed that LoF mutations in these unique PBAF complex genes sensitize tumors to PD-1 and CTLA-4 blockade [102–104]. Comparative analysis of expression and chromatin accessibility in Pbrm1-deficient cells also revealed that inactivation of *PBRM1* increases the accessibility of promoters and enhancers of IFNγ-inducible genes to transcription factors, which leads to increased expression of such genes and increased TIL levels [104].

The EZH2–PRC2 chromatin remodeling complex also plays a role in CTLA-4 blockade. Zingg et al. [103] demonstrated in melanoma mouse models that during treatment with either CTLA-4 or IL-2 blockade, TNF-α production and T cell infiltrate resulted in increased EZH2 expression, silencing tumor cell immunogenicity and antigen presentation. Inactivation of *EZH2* in this context produced a synergistic effect with CTLA-4 and IL-2, suppressing tumor growth, which suggests that EZH2 expression may serve as an immune escape mechanism during immunotherapy. The PRC2 subunit of the EZH2–PRC2 complex has been shown to cooperate with PBRM1 on PBAF complexes to repress several IFNγ-stimulated genes, providing a potential explanation as to why loss of PBAF function results in increased IFNγ-inducible gene expression [104, 105].

Another component of the BAF complex, SMARCA4, has also been implicated in driving tumor immunogenicity. In a cohort of small cell carcinoma of the ovary, hypercalcemic type tumors, LoF mutations in *SMARCA4* were highly associated with increased levels of TILs and upregulation of PD-L1 [106]. Likewise, inactivating mutations in *ARID1A* sensitized ovarian tumors to PD-L1 blockade in preclinical mouse models. A proteomic screen revealed that ARID1A interacts with the MMR gene *MSH2*, and loss of ARID1A resulted in microsatellite instability. Loss of ARID1A was also associated with increased levels of TILs and PD-L1 expression [107]. Thus, prospective mutational profiling of BAF/PBAF and EZH2–PRC2 complex genes may inform the use of ICB in the absence of other biomarkers (for example, low mutational load).

### Endogenous retroviruses

ERVs represent insertions of viral genetic material from past exogenous retroviral infections and constitute about 10% of the human genome [108], but are often silenced via epigenetic mechanisms. The use of DNA methyltransferase inhibitors [109, 110] or histone demethylase ablation [111] to increase the expression of ERV genes leads to upregulation of double-stranded RNA. Recognized as foreign viral material, such double-stranded RNA primes the innate immune system and can induce tumor cell interferon signaling and apoptosis, suggesting that derepression of ERVs could prove synergistic with ICB [110].

### Urea cycle dysregulation

The urea cycle (UC) functions to breakdown nitrogen-containing metabolites into urea, and several studies over the past decade have reported altered expression of UC genes in cancer [112–114]. Leveraging transcriptomic and ICB response data from The Cancer Genome Atlas project and three publically available melanoma studies, Lee et al. [113] found that tumors with high UC dysregulation, characterized by aberrant expression of UC genes leading to an excess of nitrogen metabolites and resultant bias for purine-to-pyrimidine transversions, were associated with better ICB response. Importantly, the resultant purine-to-pyrimidine transversion bias was a better predictor of response than TMB in these cohorts. This can be attributed to the finding that the majority of predicted neoantigens are hydrophobic, which is expected to cause stronger immunogenicity. Moving forward, UC gene expression profiles may prove to be a useful, generalizable predictor of response to ICB.

### Gut microbiome

Within the past few years, several studies have made a strong case for a link between gut microbiome composition, profiled using metagenomics, and ICB therapy outcomes. In a cohort of metastatic melanoma patients treated with PD-1 blockade, Gopalakrishnan et al. [115] identified several features of the patients’ gut microbiomes that were associated with response, including significantly higher diversity and relative abundance of *Ruminococcaceae* in responders and higher abundance of *Bacteroidales* in non-responders. CD8+ T cell abundance was found to be positively correlated with the abundance of *Faecalibacterium* and *Ruminococcaceae*, and germ-free mice receiving fecal transplants from responding patients demonstrated reduced tumor growth before therapy and improved response after therapy.

More recently, Routy et al. [116] found that patients with epithelial tumors treated with antibiotics had both shorter PFS and OS; further investigation revealed that the feces of responders were enriched in unclassified and classified Firmicutes, with *A. muciniphila* emerging as the commensal most often correlated with response. Likewise, Matson et al. [117] also observed a differential microbiome composition between PD-1 responders and non-responders in a group of metastatic melanoma patients. Further work is needed to prospectively evaluate microbiome profiling of cancer patients for patient stratification. Nevertheless, the microbiome promises to offer an exciting new set of biomarkers for improving ICB efficacy.

Though the diverse set of pathways described in this section are, at first glance, operating in different domains, they have all been demonstrated to play a role in affecting ICB response through modulation of either TIL levels or tumor immunogenicity. The interconnectedness of these apparently disparate biological features demonstrates the need for a holistic approach to stratifying patients, beyond merely one or two biological measurements (Table 2).

**Table 2:** Mechanisms of response and resistance

Pathway	Genes	Mechanism	References
TMB and neoantigen load		Low mutational load results in lack of antigenic proteins, and increased subclonal mutation/neoantigen loads are associated with poor response	[31–33, 36]
DNA damage repair	• *MSH2* • *MSH6* • *PMS2* • *POLE* • *BRCA2*	Mutations in DDR genes result in increased TMB and genomic instability, which can result in a highly antigenic and immunogenic tumor	[21, 44–46]
MAPK pathway	• *KRAS* • *STK11* • *TP53*	Oncogenic expression reduces TILs and pro-inflammatory cytokines. Activation of downstream pathways may also play a role in immunotherapy response (for example, p38 and JNK)	[57–59]
PI3K-AKT-mTOR pathway	• *PTEN*	Loss of PTEN causes oncogenic expression of PI3K pathway, which reduces TILs	[63–66]
WNT-β-catenin pathway	• *DKK2*	β-catenin suppresses chemokines that recruit DCs to the TME, and activation of *DKK2* suppresses T cells and NK cells	[71, 72]
IDO1 pathway	• *IDO1*	Expression of *IDO1* promotes activation of the PI3K pathway and immunosuppression	[73–75]
HLA variability	• *B2M*	Loss of *HLA* heterozygosity associated with decreased survival, and LoF mutations in antigen presentation genes (for example, *B2M*) can result in tumor evasion of immune response. Certain HLA supertypes are also associated with improved response (for example, HLA-B44) compared to others (for example, HLA-B62)	[76, 78, 79]
JAK-STAT pathway	• *IFNGR1* • *JAK1* • *JAK2* • *JAK3* • *ALPNR* • *SOCS1*	Lack of JAK-STAT signaling results in resistance to immunotherapy through suppression of IFNγ	[88, 90–93]
TGFβ		Expression of TGFβ enhances the function of Tregs, limiting the infiltration of T cells in the TME. TGFβ also downregulates the activity of cytotoxic lymphocytes and NK cells	[95, 96]
Chromatin remodeling	• *ARID1A* • *PBRM1* • *SMARCA4* • *EZH2*	Loss of BAF/PBAF or EZH2–PRC2 complex induces IFNγ expression. Naturally, PRC2 interacts with PBRM1 of the PBAF complex to suppress IFNγ-stimulated genes	[103–107]
Endogenous retroviruses		Upregulation of ERV genes primes the innate immune system. Several epigenetic mechanisms can increase expression of ERV genes, which leads to an elevated abundance of double-stranded RNA, and thus immune response. Such mechanisms include LoF in histone demethylases (for example, LSD1), histone deacetylases, or DNA methyltransferases	[109–111]
Urea cycle		UC dysregulation causes purine-to-pyrimidine transversion mutational bias that generates hydrophobic, highly immunogenic neoantigens	[112–114]
Microbiome		Gut microbiome composition affects T cell abundance in TME, and thus response to ICB (for example, higher levels of *Ruminococcaceae* in responders and higher levels of *Bacteroidales* in non-responders)	[115–117]

*DDR* DNA damage repair, *ERV* endogenous retrovirus, *HLA* human leukocyte antigen, *ICB* immune checkpoint blockade, *LoF* loss of function, *MHC* major histocompatibility complex, *NK* natural killer, *TIL* tumor infiltrating lymphocyte, *TMB* tumor mutational burden, *TME* tumor microenvironment, *UC* urea cycle

## Clinical implications and combination therapies

The growing repertoire of ICB studies that utilize whole-exome, whole-genome, and expression data have enabled highly specific patient stratification based on genomic and molecular aberrations. The results of these studies have shifted the focus from determining whether precision medicine is feasible to determining which biomarkers are most informative when assessing the likelihood for success of checkpoint inhibitors in a particular patient, and how to most effectively transfer this knowledge to clinical settings.

To date, only one biomarker is approved by the Food and Drug Administration (FDA) as an official criterion for ICB. Based on data from the 2015 KEYNOTE-001 trial, high expression of PD-L1 is now a requirement for use of pembrolizumab in NSCLC [118, 119]. Moreover, the FDA recently warned of an association between decreased survival and low PD-L1 expression in metastatic urothelial cancer patients who were administered pembrolizumab, which seems to reinforce the idea that high PD-L1 expression serves as a useful pan-cancer biomarker [120].

Various gene expression profiles (GEPs) are also being explored as possible predictors for ICB response. Ayers et al. [121] proposed using one such GEP for a set of immune-related genes to predict anti-PD-1 therapy response in multiple cancer types. Meanwhile, Jiang et al. [122] studied the relationship between OS and gene expression in treatment-naive patients and identified two GEPs associated with T cell dysfunction and exclusion. They were then able to leverage these GEPs to predict response to ICB in a separate cohort of melanoma patients, demonstrating their potential translational utility. To account for both the genomic and transcriptomic components of ICB response, Cristescu et al. [123] combined T cell inflammation GEP scores and TMB to predict response to pembrolizumab in a pan-cancer cohort from four KEYNOTE clinical trials and found that patients scoring high on both indicators had the strongest ORR. More research is needed to assess the stability and transferability of such gene expression biomarkers across cancer types and under different treatments.

Because of the relative cost and complexity involved in procuring and sequencing tumor samples, there has been a recent focus on finding non-invasive biomarkers. Using a blood-based assay to measure blood TMB (bTMB) from plasma circulating tumor DNA (ctDNA) instead of solid tumor, Gandara et al. [124] found bTMB to be moderately positively correlated with TMB in pre-treatment NSCLC patients. Atezolizumab-treated patients with higher bTMB had better OS and improved PFS compared to patients with lower bTMB. The presence of ctDNA in the bloodstream can be informative itself. Lee et al. [125] showed that the absence of ctDNA at baseline, or its tenfold decrease during treatment, was associated with better response and survival in metastatic melanoma patients receiving anti-PD-1 therapy. Chen et al. [126] suggested a new mechanism for tumor immune evasion via tumor shedding of PD-L1 in exosomes to suppress the immune system in metastatic melanoma patients. Higher pretreatment levels of exosomal PD-L1 were associated with poor response to pembrolizumab, suggesting that exosomal PD-L1 can be used as a biomarker for response to anti-PD-1 therapy.

However, in light of the highly variable responses seen among patients receiving ICB monotherapies—for example, many melanoma, Hodgkin lymphoma, and Merkel cell carcinoma patients do not respond to PD-(L)1 inhibitor monotherapies—treatments involving combinations of several therapies are also being explored [105]. More than 75% of the 1200 clinical trials completed by April 2017 involving PD-(L)1 inhibitors also incorporated alternative treatment modalities, including surgery, chemoradiation, small molecule inhibitors, and other checkpoint inhibitors [127].

A primary FDA-approved ICB combination therapy involves jointly administered ipilimumab and nivolumab, with recent clinical trials continuing to expand the list of cancer types for which this combination is recommended [128, 129]. In two studies analyzing anti-PD-(L)1/CTLA-4 combination therapy in NSCLC and SCLC, both of which yielded a higher ORR than PD-(L)1 monotherapy, high TMB was the main indicator for success [130, 131], possibly explaining why the combination succeeded in MMR-deficient colorectal cancer, which typically exhibits an elevated TMB. However, because anti-CTLA-4 and anti-PD-(L)1 therapies operate through complementary mechanisms at various points in the immune response, the use of TMB as a sole biomarker fails to capture the complexity of response. Thus, consideration of the effect of mutations specifically in immune-related genes and pathways affecting T cell activation and TIL concentration is imperative. Expanding on the idea of targeting multiple checkpoints simultaneously, combinations of PD-(L)1 inhibitors with alternative immune checkpoint inhibitors such as anti-TIM-3 (MBG453, NCT02608268) and anti-LAG-3 (urlumab, NCT02658981) are currently being tested in clinical trials.

Combinations of immune checkpoint therapy with targeted therapy and chemotherapy agents are also being actively investigated. The findings that BRAF and MEK inhibition therapies can lead to increased PD-L1 expression within tumors suggest that the efficacy of such therapies might be augmented by joint administration with PD-(L)1 inhibition [132]. Increased immunogenicity of T cells is a side effect of BRAF, MEK, and VEGF inhibition monotherapies [54, 133–136], with promising early results from trials testing combined VEGF and PD-(L)1 blockade [137]. More recently, results from the KEYNOTE-189 trial indicated that in certain NSCLC patients, the combination of pembrolizumab with standard pemetrexed and platinum-based chemotherapy led to improved OS and PFS [127]. However, highlighting the complexity and unpredictability of multidrug interactions, in the KEYNOTE-252 clinical trial, which combined pembrolizumab with an IDO1 inhibitor, the dual drug approach did not significantly improve PFS over pembrolizumab monotherapy, and subsequently the trial was halted [138]. In a recent trial [139], MEKi/anti-PD-L1 combination therapy also failed to meet its primary endpoint in patients with microsatellite-stable/microsatellite instability-low metastatic colorectal cancer. However, results from clinical trials testing MEKi/ICB combination therapy in other tumor types have yet to be reported. An increase in adverse effects is also a potential drawback of drug combinations: an early trial combining CTLA-4 and BRAF inhibitors was ended early due to a high rate of hepatic adverse events [140].

Epigenetic therapies are also strong candidates for use alongside ICB. Inhibitors of DNA methyltransferase, histone deacetylase, and histone demethylase (for example, LSD1) play an immunostimulatory role, operating via potentiation of T cells, induction of cytokine production, inhibition of Tregs, or upregulation of antigen presentation [141, 142]. Currently, several clinical trials are exploring the possible synergies between ICB and epigenetic inhibitors such as DNA methyltransferase inhibitors 5-azacytidine and histone deacetylase inhibitors Vorinostat, among others [141].

Finally, combinations of ICB with radiation therapy are also under investigation, based on the theory that a patient’s immune system is activated following radiation-induced malignant cell death and subsequent inflammation. This has proven to be the case even at sites distal to the original target of radiation, a phenomenon often referred to as the “abscopal effect” [143]. The combination of focal radiation with anti-CTLA-4 was recently demonstrated to induce an immune response in chemo-refractory metastatic NSCLC [144], but in general, more testing is needed to reach a consensus on optimal radiation dosage when utilized in tandem with ICB [145].

## Conclusions and future directions

The advent of ICB has been a watershed moment in the treatment of cancer. Certain cancers that corresponded to a death sentence a mere two decades ago are now readily treatable in a significant fraction of patients, which in some cases may result in complete remission. Alterations in the pathways and mechanisms described in this review have the potential to join traditional biomarkers such as TMB and PD-L1 expression as ways of stratifying patients to maximize ICB efficacy. Even so, as evidenced by the uncertainty surrounding the heterogeneity of responses across cancer types and even across patients with similar tumors, there are still many aspects of the immune–tumor interaction that have yet to be fully characterized before these new therapies can be applied optimally.

Cytotoxic T cells are not operating in isolation, and the concentrations of other cell types in the TME, such as suppressive Tregs and stimulatory T-helper cells, also affect the efficacy of ICB. Histological analysis to determine TIL levels can inform decision-making, and part of the reason combination therapies have been successful is because immunotherapies can themselves alter the composition of cells infiltrating the tumor: blocking CTLA-4 in particular elicits an increase in COS+ Th1-like CD4 effector cells in the TME [146]. Additionally, the role the innate immune system plays in potentiating the anti-tumor immune response is more important than previously realized, as demonstrated not only in the context of ERV-induced upregulation of interferon, but also by recent findings regarding the effect of immune checkpoint therapy on NK cells in the TME. Blockade of the T cell immunoreceptor with Ig and ITIM domains (TIGIT) costimulatory receptor, found on both T cells and NK cells, has been found to alleviate NK cell exhaustion and boost the anti-tumor immune response to PD-(L)1 blockade, with NK cell presence in the TME necessary for the effects of TIGIT or PD-(L)1 blockade [147].

Integration of novel methods and technologies into ICB response research will add to an understanding of its biological underpinnings. Wider use of and advances in single cell sequencing will enable a finer mechanistic understanding of the multifactorial interactions affecting T cell activity in the TME. Most studies aimed at finding genomic correlates of response have strictly utilized whole-exome sequencing. However, exomes only capture approximately 1% of the genome, and expanding these analyses into the whole-genome space will enable the identification of relevant alterations in regulatory regions, such as promoters and enhancers, and structural variants [148]. Activation and inactivation of cancer genes is not limited to mutations and structural variants, and incorporating epigenomic data (for example, methylation) also has the potential to reveal further significant biological associations with ICB response. For example, mutational signature analysis in breast cancers revealed that promoter methylation of *RAD51C* had a similar effect on HR deficiency as biallelic inactivation of *BRCA1/2* [149]. Additionally, long read sequencing technology will enable more accurate identification of alternatively spliced transcripts, which may be associated with response.

Lastly, the genomics underlying the variability in irAEs across patients are also not yet well understood. Though in serious cases irAEs may require early discontinuation of immune checkpoint therapy, they can also serve as a sign of immune potentiation and potentially efficacy. For example, a recent study in NSCLC found that earlier onset of irAEs is associated with higher ORR for PD-(L)1 blockade [150]. Understanding whether it will be possible to maintain similar levels of immunotherapy response in such patients while curtailing the incidence of irAEs will require additional exploration at the genomic and molecular levels.

The current resources being invested in checkpoint inhibitor development and clinical trials ensure that our understanding of immunotherapeutic drugs will continue to grow in the near future. It is likely that checkpoint inhibitors will ultimately not prove a silver bullet, but a powerful new arrow in the growing quiver of cancer therapies.

## Abbreviations

APC: Antigen-presenting cell
BAF: BRG1-associated factor
bTMB: Blood tumor mutational burden
ctDNA: Circulating tumor DNA
CTLA-4: Cytotoxic T lymphocyte-associated antigen 4
DC: Dendritic cell
DDR: DNA damage repair
ERK: Extracellular signal–regulated kinase
ERV: Endogenous retrovirus
FDA: Food and Drug Administration
GEP: Gene expression profile
HLA: Human leukocyte antigen
HR: Homologous recombination
ICB: Immune checkpoint blockade
IDO1: Indoleamine 2,3-dioxygenase 1
irAE: Immune-related adverse effect
ITH: Intratumor heterogeneity
JAK-STAT: Janus kinase/signal transducers and activators of transcription
LoF: Loss of function
LoH: Loss of heterozygosity
LUAC: Lung adenocarcinoma
MAPK: Mitogen-activated protein kinase
MEKi: MAPK/ERK kinase inhibitors
MHC I/II: Major histocompatibility complex class I/II molecules
MMR: Mismatch repair
NK: Natural killer
NSCLC: Non-small cell lung cancer
ORR: Objective response rate
OS: Overall survival
PBAF: Polybromo-associated BAF
PD-(L)1: Programmed death ligand 1
PD-1: Programmed cell death protein 1
PFS: Progression-free survival
PTEN: Phosphatase and tensin homolog
TCR: T cell receptor
TGFβ: Transforming growth factor beta
TIL: Tumor infiltrating lymphocyte
TMB: Tumor mutational burden
TME: Tumor microenvironment
Treg: Regulatory T cell
UC: Urea cycle
VEGF: Vascular endothelial growth factor
